# A McLafferty-Type Rearrangement in the Pentafluorobenzyl Ester Derivatives of Dialkyl Phosphates in GC-NICI-MS/MS: A Mini-Review and a Meta-Analysis

**DOI:** 10.3390/molecules31010022

**Published:** 2025-12-21

**Authors:** Dimitrios Tsikas

**Affiliations:** Core Unit Proteomics, Institute of Toxicology, Hannover Medical School, 30623 Hannover, Germany; tsikas.dimitros@mh-hannover.de

**Keywords:** collision-induced dissociation, electron ionization, ionization, mass spectrometry, mechanisms, negative-ion chemical ionization, organophosphates

## Abstract

Rearrangements such as the McLafferty rearrangement are common in mass spectrometry of positive ions, but rare in negative ions. The present article searched the literature for McLafferty rearrangements of negative ions formed by negative electrospray ionization (-ESI) or by negative-ion chemical ionization (NICI). McLafferty Rearrangements have been reported for negative ions formed by -ESI, yet not for anions formed by NICI in GC-MS and GC-MS/MS. This article reports for the first time a McLafferty Rearrangement of the collision-induced dissociation (CID) of precursor ions due to [M−PFB]^−^ generated by NICI in the GC-MS/MS analysis of pentafluorobenzyl (PFB) esters and thioesters of diethyl phosphates (CH_3_CH_2_O)_2_PO_2_, diethyl thiophosphates (CH_3_CH_2_O)_2_PSO, and diethyl dithiophosphates (CH_3_CH_2_O)_2_PS_2_. Two specific product anions due to α,β-cleavages and formation of neutral losses of ethene and ethanol are formed. No McLafferty rearrangements were observed in the CID of 1-chloropropyl derivatives of these dialkyl organophosphates in the positive-ion chemical ionization (PICI) mode. McLafferty rearrangements of PFB esters of organophosphates have been observed in the EI mode. No McLafferty rearrangements were observed in the EI and NICI of diethyl fluoro phosphate, a representative of dialkyl fluoro phosphates. McLafferty rearrangements of negative ions seem to be more common than assumed so far.

## 1. Introduction

**Scheme 1:**
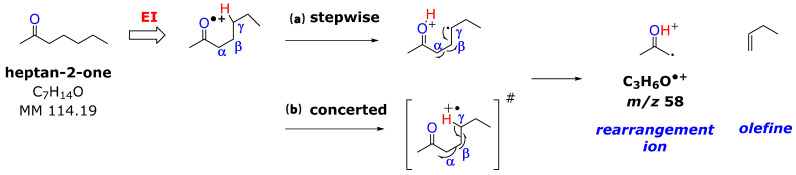
Stepwise and concerted McLafferty rearrangement mechanisms of the electron ionization (EI) of the model molecule heptan-2-one. MM, molecular mass. Constructed according to Laulhé et al. 2012 [2].

According to the *IUPAC Compendium of Chemical Terminology*, the McLafferty rearrangement in mass spectrometry describes a,β-cleavage with concomitant specific transfer of a γ-hydrogen atom in a six-membered transition state in mono-unsaturated systems, irrespective of whether the rearrangement is formulated by a radical or an ionic mechanism, and irrespective of the position of the charge (IUPAC 2025) [1]. A typical example for a McLafferty rearrangement is provided in Scheme 1 for the stepwise and concerted electron ionization (EI) of heptan-2-one (C_7_H_14_O; MM, 114.19), which generates the rearrangement radical cation with *m*/*z* 58 and the neutral loss but-1-ene (MM, 56.11) [2].

The McLafferty rearrangement is known to occur with a reverse charge distribution, i.e., generating the neutral enol and the charged alkene fragments, such as in hexanal [3]. The McLafferty rearrangement and its variants have been described in detail in books on mass spectrometry [4,5,6] and in scientific data banks such as PubMed (https://pubmed.ncbi.nlm.nih.gov). The vast majority of published McLafferty rearrangements refer to positive ions generated by EI. Extremely rare are published articles reporting on McLafferty rearrangements for negative ions observed in-source by negative-ion chemical ionization (NICI), or by collision-induced dissociation (CID) of precursor anions in collision cells of mass spectrometers such as gas chromatography-tandem mass spectrometry (GC-MS/MS) instruments.

**Scheme 2:**
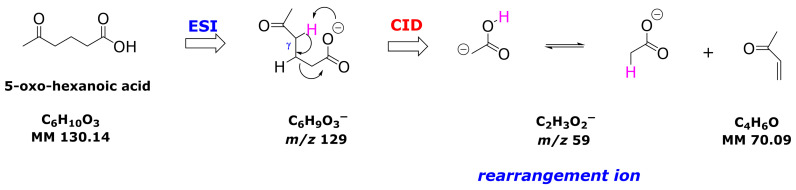
McLafferty rearrangement mechanisms of the precursor ion with *m*/*z* 129 generated by negative electrospray ionization of 5-oxo-hexanoic acid according to Grossert et al. 2006 [7], after modification by the author of the present work. CID, collision-induced association; ESI, electrospray ionization; MM, molecular mass.

McLafferty rearrangements have been reported on negative ions produced by electrospray ionization (ESI) and tandem mass spectrometry (MS/MS) both in ion trap and triple quadrupole mass spectrometers of carboxylate anions with acidic γ hydrogen atoms such as 5-oxo-hexanoic acid with a prominent anion at *m*/*z* 59, presumably due to acetate [CH_3_COO]^−^ produced by CID of the precursor [M−H]^−^ at low collision energy values (e.g., 5 eV) (Scheme 2) [7].

**Scheme 3:**
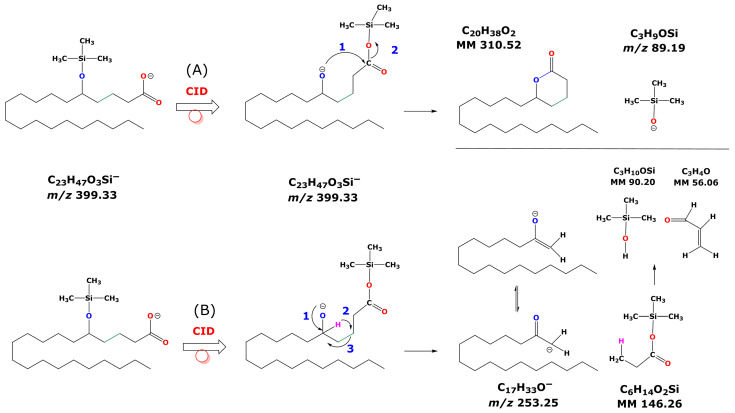
Schematic of the Murphy rearrangement originally described by de Laclos et al. 1993 [9], here illustrated for 5-hydroxyeicosanoic acid. Upon TMS ether-to-TMS ester rearrangement of the precursor with *m*/*z* 399.3, two reactions may occur. (**A**) The hydroxylate anion on C-5 attacks the carboxyl ester group to form the neutral loss 310.5 (a δ-lactone) and the ion with *m*/*z* 89 TMSO^−^. (**B**) The hydroxylate anion on C-5 attacks the C-5 to form the anion with *m*/*z* 253 and the neutral loss 146, which decomposes to acrolein (MM 56) and TMSOH (MM 90). CID, collision-induced dissociation; MM, molecular mass; TMS, trimethylsilyl; TMSOH, trimethylsilanol.

The McLafferty rearrangement is the most prominent gas-phase rearrangement in mass spectrometry. We may assume that numerous less common and still anonymous rearrangements remain to be discovered and named. Recently, the name Murphy rearrangement has been proposed [8] for a rearrangement reported by Murphy’s group [9]. In brief, the NICI of pentafluorobenzyl (PFB) ester trimethylsilyl (TMS) derivatives of catalytically desulfurized and saturated metabolites of arachidonic acid of the 5-lipoxygenase pathway, i.e., cysteinyl leukotrienes and leukotriene B_4_, generates carboxylate anions due to [M−PFB]^−^, the CID of which forms highly specific rearrangement product anions. The mechanism of the Murphy rearrangement involves a six-membered transition state analogous to typical McLafferty rearrangements observed under EI conditions. In the case of the Murphy rearrangement of 5-hydroxyeicosanoic acid, the rearrangement ion is an anion, and the neutral loss is acrolein, an olefinic aldehyde (Scheme 3).

## 2. Methods

These observations prompt us to search the literature for potential McLafferty rearrangements in the NICI mode that might have occurred in the ion-source or in collision cells of GC-MS and GC-MS/MS instruments. We were especially interested in PFB ester derivatives of acidic compounds such as organic carboxylic acids and organic (thio)phosphates, which are accessible for derivatization with PFB bromide (PFB-Br). PFB-Br has been widely used for the derivatization of organic and inorganic substances, which generally readily ionize to negative ions [10,11].

Several groups have analyzed dialkyl phosphates and related substances, including the dialkyl fluoro phosphates (DAFPs), by GC-MS- and LC-MS-based methods [12,13,14,15,16,17,18,19,20,21,22,23,24,25]. In the context of lithium-ion batteries, a fraction of the discovered compounds was found to belong to the group of fluorophosphates (phosphorofluoridates), such as diisopropyl fluorophosphates, which are suspected of potential toxicity [24,25]. For an extended review of dialkyl phosphates in the area of metal-organic framework (MOF), see the article by Ryu and colleagues [26]. It is worth mentioning that dialkyl fluorophosphates, including diethyl fluoro phosphate (DEFP), are volatile, and their GC-MS analysis does not require any derivatization.

Oglobline and colleagues investigated the GC-MS and GC-MS/MS analysis of PFB esters of a series of dialkyl phosphates (DAPs) in the NICI mode [13]. Schindler and colleagues [21] investigated the CID of the PFB derivatives of *m*- and *p*-cresyl phosphates in the EI mode. In the present work, the data reported by Oglobline et al. [13] and Schindler et al. [21] were examined with regard to possible McLafferty rearrangements. On the recommendation of an anonymous reviewer of the present work, mass spectrometry data reported on dialkyl fluoro phosphates (DAFPs) were included in the study. The data reported by the group of Nowak [24,25] were also considered, although these substances had been analyzed directly, i.e., without any derivatization.

Information relating to NICI and CID conditions used by the authors of the examined papers is provided in the Results and Discussion section as originally reported.

The chemical structures and the names of the investigated molecules were drawn and suggested by ChemDraw 15.0 Professional (PerkinElmer, Hamburg, Germany).

## 3. Results and Discussion

### 3.1. McLafferty Rearrangement in PFB Derivatives of Organophosphates in the NICI Mode [13]

The DAP investigated by Oglobine et al. [13] included dimethyl phosphate (DMP), diethylphosphate (DEP), dimethyl thiophosphate (DMTP), diethyl thiophosphate (DETP), dimethyl dithiophosphate (DMDTP), and diethyl dithiophosphate (DEDTP). Dibutyl phosphate (DBP) was used as an internal standard in quantitative analyses. Oglobline et al. reported the most intense ions found in the GC-MS mass spectra, i.e., [M−PFB]^−^, and the two most specific product ions produced by CID of these precursor ions. These data are summarized in Table 1. Unfortunately, no data had been reported for dibutyl phosphate by Oglobline et al. [13].

Analyses were carried out on a Finnigan/MAT TSQ 46 GC-MS/MS (San Jose, CA, USA) in the NICI mode using methane (120 Pa) as the reagent gas. The optimal ion-source temperature for the most abundant product ion formation was 140 °C. Argon was used as the collision gas at a cell pressure of 0.2 Pa. Optimal collision energy values were reported to be 10–15 eV. The retention time values of the PFB esters were 9.20 min for DMP, 11.10 min for DEP, 12.00 min for DMTP, 13.20 min for DMDTP, 13.34 min for DETP, 14.42 min for DEDTP, and 16.06 min for DBP.

**Scheme 4:**
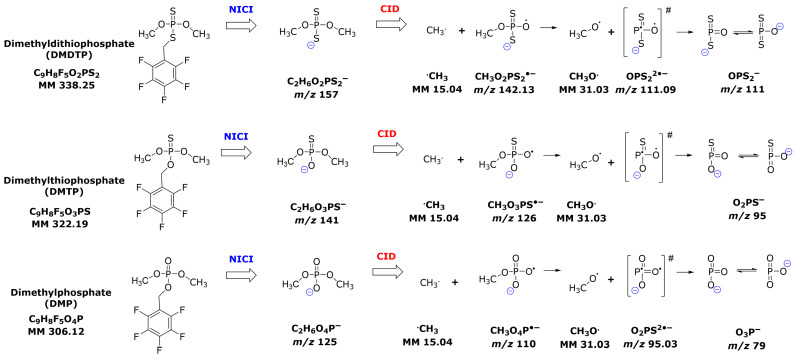
Proposed structures for the PFB ester derivatives of DMDTP, DMTP and DMP, the most intense ions formed by NICI, i.e., [M−PFB]^−^, of the product ions, and the corresponding neutral loss species ^●^CH_3_ and CH_3_O^●^. The structures in brackets are potential intermediates in transitional states, indicated by [] that contain the structure of the ion and by the symbol #. NICI, negative-ion chemical ionization; CID, collision-induced dissociation; MM, molecular mass. Constructed with data reported by Oglobine et al. [13]. See also Table 1.

**Scheme 5:**
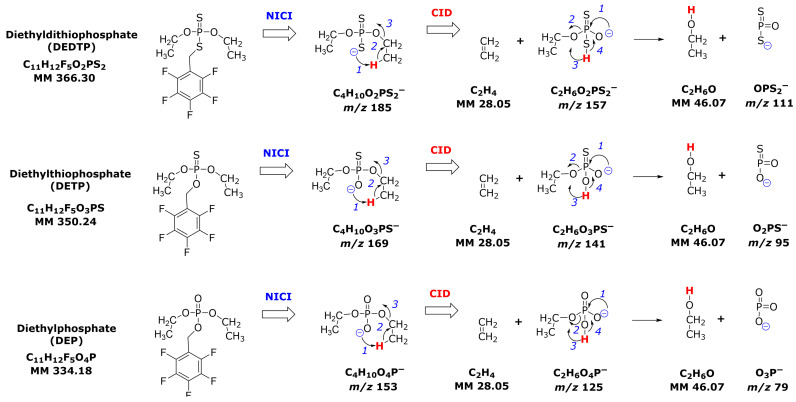
Proposed structures for the PFB ester derivatives of DEDTP, DETP and DEP, the most intense precursor ions formed by NICI, i.e., [M−PFB]^−^, a McLafferty-type rearrangement for the formation of the two product ions, and the corresponding neutral loss species CH_2_=CH_2_ and CH_3_OH. NICI, negative-ion chemical ionization; CID, collision-induced dissociation; MM, molecular mass. Numbers on the arrows indicate the sequence of the steps. Constructed with data reported by Oglobline et al. [13]. See also Table 1.

PFB esters of dialkyl phosphates are expected to ionize to their dialkyl phosphate anions by losing their PFB moieties. The *m*/*z* values of the precursor ions with [M−PFB]^−^ are all ODD, and the number of their N atoms is zero, i.e., EVEN. According to the nitrogen rule in NICI [27], all precursor ions [M−PFB]^−^ have a visible negative charge, which is most likely located on the O and S atoms as shown in Scheme 4 and Scheme 5.

The purely inorganic product ions, i.e., ions 2 in Table 1, have all ODD *m*/*z* values, which means that they carry a visible negative charge on their O or S atoms. The organic product ions, i.e., ions 1 in Table 1, have ODD *m*/*z* values in the diethyl DAP, but they have EVEN *m*/*z* values in the dimethyl DAP. Thus, the product ions 1 of the dimethyl DAP are distonic radical anions. It is notable that organic phosphates isomerize to distonic structures in the gas phase in the EI mode [28].

These observations suggest that the precursor ions [M−PFB]^−^ of the dimethyl DAP and diethyl DAP dissociate by distinctly different mechanisms in the collision cell. It remains to be examined whether the CID of [M−PFB]^−^ of the diethyl DAP, that is associated with the formation of the olefine CH_2_=CH_2_, is a McLafferty or McLafferty-type rearrangement.

**Scheme 6:**
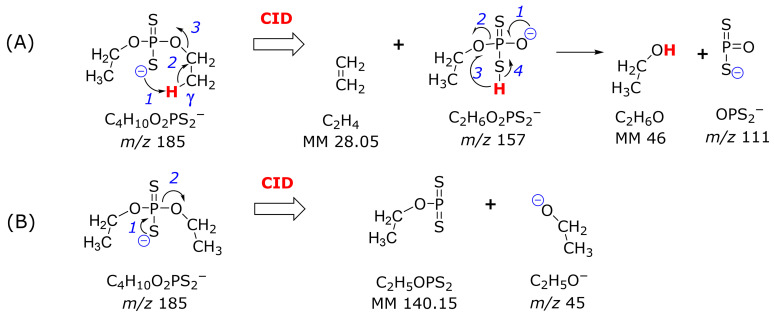
Comparison of two mechanisms for the CID of the precursor ion [M−PFB]^−^ with *m*/*z* 185 from the NICI of the PFB ester derivative of DEDTP. (**A**) Formation of the product ions with *m*/*z* 157 and *m*/*z* 111, and the neutral losses of 28 and 46. (**B**) Formation of the product ion with *m*/*z* 45 and the neutral loss of 140. CID, collision-induced dissociation; MM, molecular mass. Mechanism (**A**) was constructed with data reported by Oglobine et al. [13]. Mechanism (**B**) is not supported by data from Oglobine et al. [13]. See also Table 1.

Scheme 6 compares two mechanisms for the CID of the precursor ion [M−PFB]^−^ with *m*/*z* 185 from the NICI of the PFB ester derivative of DEDTP. Mechanism (A) results in the formation of the product ions with *m*/*z* 157 and *m*/*z* 111 and the neutral losses of 28 and 46. Mechanism (B) would form the product ion with *m*/*z* 45 and the neutral loss of 140. The data reported by Oglobine et al. [13] support mechanism (A) but not mechanism (B).

### 3.2. McLafferty Rearrangement in PFB Derivatives of Organophosphates in the EI Mode [21]

**Scheme 7:**
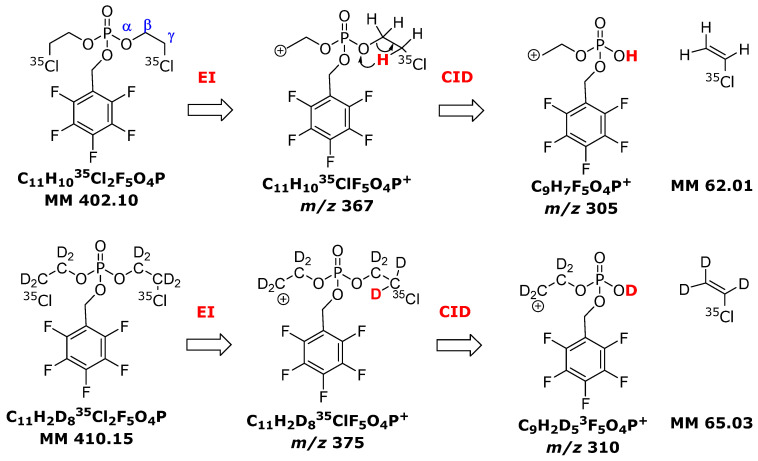
EI of the PFB ester derivatives of unlabeled and deuterium-labeled *bis*(2-chloroethyl)phosphate to their precursors [M−^35^Cl]^+^ and their CID to the rearrangement ions and the neutral loss vinyl chloride. CID, collision-induced dissociation; EI, electron ionization; MM, molecular mass. Constructed with data reported by Schindler et al. 2014 [21].

Examination of the data reported by Schindler and colleagues [21] revealed no McLafferty-like rearrangement in the CID of the PFB derivatives of *m*- and *p*-cresyl phosphates in the EI mode [21]. In contrast, the PFB ester derivative of *bis*(2-chloroethyl)phosphate underwent a CID-McLafferty-type rearrangement in the EI mode, apparently without the participation of the PFB moiety, yet with a transfer of one of the two γ H (D) atoms from the chloroethyl group to the S atom of the phosphate group (Scheme 7).

Schindler and colleagues [19] reported on the quantitative determination of PFB derivatives of DBP (MM, 390.29) and DBP-d_18_ (MM, 408.40) by GC-MS/MS, yet without specification of the ionization mode [19]. The reported precursor ions at *m*/*z* 390, *m*/*z* 346, and *m*/*z* 335 and the product ions at *m*/*z* 279, *m*/*z* 181, *m*/*z* 259, and *m*/*z* 282 [19] suggest that EI was used. Ueyama et al. [29] analyzed DBP by GC-MS in the EI mode and reported a single ion with *m*/*z* 335. These data do not allow a reliable examination of a McLafferty rearrangement in the PFB derivative of DBP.

**Scheme 8:**
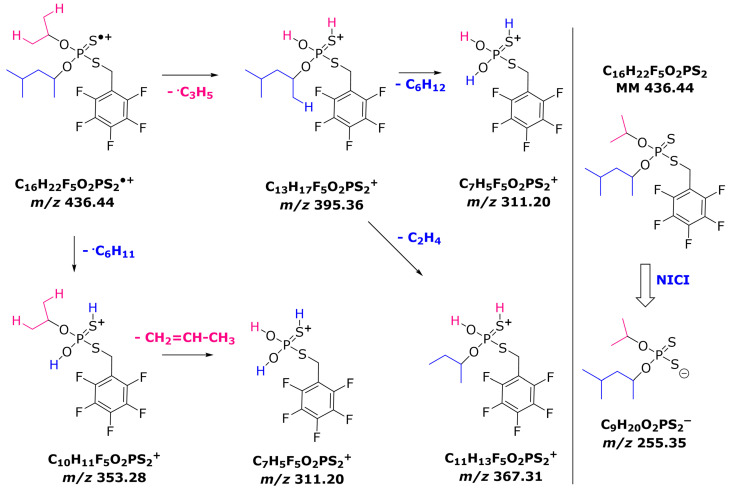
Proposed structures for the PFB ester derivative of *O*-isopropyl *O*-(4-methylpentan-2-yl) *S*-((perfluorophenyl)methyl) phosphorodithioate (C_16_H_22_F_5_O_2_PS_2_, MM 436.44) and of the ions in its electron ionization GC-MS mass spectrum. γ H atoms are assumed to be transferred from the alkyl moieties that are shown in different colors (magenta and blue, respectively). MM, molecular mass. Constructed with data reported by Becchi et al. [30].

Becchi and colleagues reported on the GC-MS analysis of PFB derivatives of numerous dithio DAP in the EI (70 eV; ion source, 230 °C) and the NICI (methane; 136 eV; ion source, 150 °C) mode, notably the *m*/*z* values of the [M−PFB]^−^ ions [30]. These data do not allow examination of McLafferty-type rearrangements in the PFB derivatives in the NICI mode. In the EI mode, cleavages of one and two alkyl radicals were reported to occur. In the reported GC-MS mass spectrum of the PFB derivative of *O*-isopropyl *O*-(4-methylpentan-2-yl) *S*-((perfluorophenyl)methyl) phosphorodithioate (C_16_H_22_F_5_O_2_PS_2_, MM 436.44) the following *m*/*z* (approximate intensity, %) ions were found: 395 (1), 367 (17), 311 (100), 181 (60); 353 (40), 311 (90), 181 (100). The alkyl chains of this derivative have several γ H atoms, which are likely to have been transferred to the O and S atoms upon EI (Scheme 8). Becchi et al. [30] did not report on CID of the PFB derivatives in EI or NICI.

### 3.3. Dialkyl Fluoro Organophosphates [24]

Weber and colleagues reported on the identification of dialkylated fluoro phosphates by GC-MS using EI, PICI and NICI of a thermally aged commercial lithium-ion battery electrolyte [24]. In the present work, the data reported by Weber et al. [24] were examined with respect to McLafferty rearrangements in the EI and NICI modes. In this context, the study by Weber et al. [24] did not report on such investigations and has limitations for the present study, because no mass spectra of synthetic compounds were presented. Also, no CID experiments had been performed [24].

**Scheme 9:**
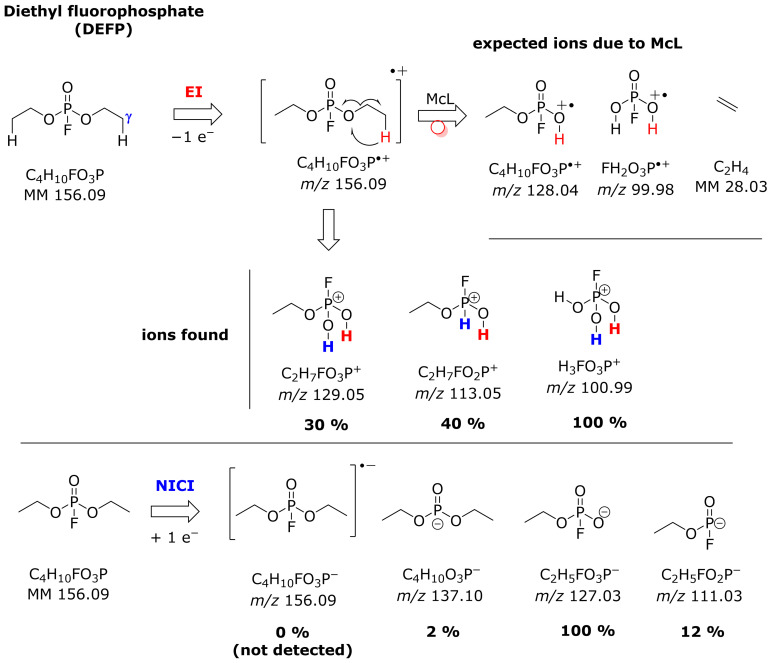
Proposed structures for diethyl fluoro phosphate (C_4_H_10_FO_3_P, MM 156.09) and ions in its electron ionization (EI) and negative-ion chemical ionization (NICI) GC-MS mass spectra. In the McLafferty (McL) rearrangement in the EI mode, γ H atoms are assumed to be transferred from the ethyl moieties to the O atoms of the phosphate group. In the EI mode, expected and measured ions are reported in the case of a McLafferty rearrangement. In the NICI mode, found ions and their intensity are reported. The blue- and red-colored H atoms indicate different origins. MM, molecular mass. Constructed with data reported by Weber et al. [24].

Weber et al. reported that in the NICI mass spectra of the clearly identified compounds, none of the formed ions resulted from a C-C-bond cleavage [24]. Scheme 9 shows proposed mechanisms for the EI and NICI of diethyl fluoro phosphate (DEFP) as an example of a compound that possesses γ H atoms, i.e., with the potential to undergo McLafferty rearrangements. Unlike the PFB ester of DEP and DETP, DEFP does not seem to undergo a McLafferty rearrangement in the NICI mode.

In the EI, McLafferty rearrangement is expected to form the rearrangement ions with *m*/*z* 128 and *m*/*z* 100 and the olefine C_2_H_4_ (MM, 28). A molecular radical cation at *m*/*z* 156 was not observed. In fact, the ions found by Weber et al. were *m*/*z* 129, *m*/*z* 113 and *m*/*z* 101 (base peak) [24]. The present work suggests that DEFP does not undergo a McLafferty rearrangement under EI conditions (Scheme 9), yet, as mentioned above, the data reported by Weber et al. are not based on the analysis of synthetic DEFP, but on data from thermal degradation of DEFP and other investigated dialkyl fluoro phosphates [24].

## 4. Conclusions

The McLafferty rearrangement is one of the most prominent rearrangements in mass spectrometry under electron ionization (EI) conditions. The McLafferty rearrangement also occurs in the CID of the precursor [M−Cl]^+^ of the PFB ester derivative of 3-chlorodiethyl phosphate. The present work reports the first McLafferty rearrangement of the CID of the precursors [M−PFB]^−^ of the PFB derivatives of diethyl, diethylthio and diethyldithio phosphates during their GC-MS/MS analysis in the negative-ion chemical ionization (NICI) mode. In the diethyl fluoro phosphate, no McLafferty rearrangements were observed in the EI and NICI modes. The few examined studies on PFB ester derivatives of organic phosphates suggest that McLafferty rearrangement and McLafferty-type rearrangements could be more common in the gas-phase chemistry of anions than previously assumed.

## Figures and Tables

**Table 1:** Dialkyl phosphates (DAPs) as PFB derivatives, their precursor ions [M−PFB]^−^, and two major product ions. The H in bold in Ion 1 derives from the diethyl group. The Table was constructed with data reported by Oglobline et al. [13].

DAP	Precursor Ion		Product Ions			
	[M−PFB]^−^	*m*/*z*	Ion 1	*m*/*z*	Ion 2	*m/z*
DMP	[(CH_3_O)_2_PO_2_]^−^	125	[(CH_3_O)PO_3_]^−^	110	[PO_3_]^−^	79
DEP	[(C_2_H_5_O)_2_PO_2_]^−^	153	[(C_2_H_5_O)**H**PO_2_]^−^	125	[PO_3_]^−^	79
DMTP	[(CH_3_O)_2_PSO]^−^	141	[(CH_3_O)PO_2_S]^−^	126	[PO_2_S]^−^	95
DETP	[(C_2_H_5_O)_2_PSO]^−^	169	[(C_2_H_5_O)**H**PO_2_S]^−^	141	[PO_2_S]^−^	95
DMDTP	[(CH_3_O)_2_PS_2_]^−^	157	[(CH_3_O)POS_2_]^−^	142	[POS_2_]^−^	111
DEDTP	[(C_2_H_5_O)_2_PS_2_]^−^	185	[(C_2_H_5_O)**H**POS_2_]^−^	157	[POS_2_]^−^	111

## Data Availability

The study did not report any data.
